# Modifications of Polymeric Membranes Used in Guided Tissue and Bone Regeneration

**DOI:** 10.3390/polym11050782

**Published:** 2019-05-02

**Authors:** Wojciech Florjanski, Sylwia Orzeszek, Anna Olchowy, Natalia Grychowska, Wlodzimierz Wieckiewicz, Andrzej Malysa, Joanna Smardz, Mieszko Wieckiewicz

**Affiliations:** 1 Department of Experimental Dentistry, Faculty of Dentistry, Wroclaw Medical University, 50-367 Wroclaw, Poland; wojtek.florjanski@gmail.com (W.F.); sylwia.winiewska87@gmail.com (S.O.); ania.olchowy@gmail.com (A.O.); andrzejmalysa@o2.pl (A.M.); joannasmardz1@gmail.com (J.S.); 2 Department of Prosthetic Dentistry, Faculty of Dentistry, Wroclaw Medical University, 50-367 Wroclaw, Poland; natgrychowska@gmail.com (N.G.); protetyka.stom@umed.wroc.pl (W.W.)

**Keywords:** guided tissue regeneration, guided bone regeneration, membrane, modification, coating

## Abstract

Guided tissue/bone regeneration (GTR/GBR) is a widely used procedure in contemporary dentistry. To achieve the required results of tissue regeneration, soft tissues that reproduce quickly are separated from the slow-growing bone tissue by membranes. Many types of membranes are currently in use, but none of them fulfil all of the desired features. To address this issue, further research on developing new membranes with better separation characteristics, such as membrane modification, is needed. Many of the current innovative modified materials are still in the phase of in vitro and experimental studies. A collective review on new trends in membrane modification to GTR/GBR is needed due to the widespread use of polymeric membranes and the constant development in the field of dentistry. Therefore, the aim of this review was to present an overview of polymeric membrane modifications to the GTR/GBR reported in the literature. The authors searched databases, including PubMed, SCOPUS, Web of Science, and OVID, for relevant studies that were published during 1999–2019. The following keywords were used: guided tissue regeneration, membranes, coating, and modification. A total of 17 papers were included in this review. Furthermore, the articles were divided into three groups that were based on the type of membrane modification: antibiotic coating, ion-use modifications, and others modifications, thus providing an overview of current existing knowledge in the field and encouraging further research. The results of included studies on modified barrier membranes seem to be promising, both in terms of safety and benefits for patients. However, modifications result in a large spectrum of effects. Further clinical studies are needed on a large group of patients to clearly confirm the effects that were observed in animal and in vitro studies.

## 1. Introduction

Modern dental treatment in the field of maxillofacial and oral surgery, implantology, and periodontics widely uses guided tissue/bone regeneration (GTR/GBR) [1,2,3]. To rapidly and efficiently regenerate tissues, a number of methods for securing the augmentation material by using membranes have been developed [4,5]. Tissue healing process depends on two main objectives. The first one is the stimulation of the growth of surrounding tissues and the second one is the prevention of the growth and proliferation of epithelial and connective tissue cells [6,7]. GTR/GBR is a procedure necessary for separating proliferating soft tissues that reproduce very quickly from bone tissue, which grows more slowly—a process that is termed cell exclusion [8,9].

The currently used GTR/GBR membranes are subjected to a number of requirements. They should be biocompatible, safe, non-allergenic, non-toxic, and mechanically stable, and this stability should allow for maintaining tissue dimension [10]. The rate of degradation should also be appropriate. For bone reconstruction, the membrane should function as a barrier for 3–4 weeks. The membranes should also be able to integrate with tissues, transfer nutrients, and increase tissue adhesion. Meeting all of these criteria helps in achieving the success of the procedure. None of the commercially available membranes meet all of these criteria.

The currently used membranes can be divided into resorbable and non-resorbable membranes. On the one hand, non-resorbable membranes have the perfect ability to maintain the space and shape of the cavity; they are also mechanically stable, so that they inhibit cell migration process. On the other hand, the use of non-resorbable membranes requires additional procedure, which could remove them and thus interfere with the healing process [11]. Among the non-resorbable membranes, polytetrafluoroethylene (PTFE) and titanium-reinforced PTFE are the most commonly implanted. Resorbable membranes are created to avoid the need for repeated surgery and at the same time reduce the risk of complications [12,13]. They have some biogenic properties that accelerate tissue healing. Resorbable membranes also have limitations despite unquestionable advantages. The process of degradation of resorbable membranes may be associated with the formation of local inflammatory reactions, which may undoubtedly adversely affect the healing process [14,15]. Resorbable membranes can be made from natural or synthetic materials. Natural materials have high biocompatibility and high bioactivity, but they degrade faster. Synthetic materials have more predictable degradation, but they can initiate the hypersensitivity and allergic reaction. The most common natural resorbable materials are collagen, chitosan, and gelatine [16,17,18]. The most common synthetic resorbable materials are organic, aliphatic thermoplastic polymers, such as poly(lactic acid), poly(glycolic acid), and their copolymers [19]. Despite the advantages and ease of use, all of the above-mentioned membranes still do not meet all of the clinical requirements, which results in the constant need to modify them. Many of the research methods for membrane modifications are still in the phase of in vitro studies, which makes their potential clinical use difficult. Due to the wide use of polymeric membranes and the constant development in the field of dentistry, there is a need to create a collective study, in which new trends in membrane modifications to the GTR/GBR are presented.

In this scientific review, modern methods of membrane modification and their new properties are presented. To the best of authors’ knowledge, this topic has not been discussed in a similar way earlier. Therefore, the aim of this review was to present a collection of studies regarding polymeric membrane modifications to the GTR/GBR with the determination of future potential directions of development in this field. The modification methods that are described below are designed to change the negative properties, improve advantages, or introduce completely new features of materials, from which the popularly available membranes are made.

## 2. Materials and Methods

The authors searched databases, including the PubMed, SCOPUS, Web of Science, and OVID for relevant papers that were published during 1999–2019. The selected time range, that is, the last 20 years, is associated with the intense development of technologies that are related to GBR. The following keywords were used: guided tissue regeneration, membranes, coating, and modification. Medical Subject Headings were used to develop a literature search strategy, as follows: the phrase (1) “Guided tissue regeneration”, was combined with each of the following phrases: (a) “membranes”, (b) “coating”, (c) “modification”, viz. (1) + (a),“guided tissue regeneration membranes”, (2) + (b), “guided tissue regeneration coating”, etc. Original papers published in English focusing on modifications of membranes that were used for GTR/GBR, which were significant from the modern clinical viewpoint and practical validity, were considered. The final selection was based on a comprehensive description of methodology and the clearness of reporting of all the articles that were found, according to the initial criteria. The reference list of included articles was also screened to identify other potentially appropriate studies.

## 3. Results

The first selection of potential studies was conducted based on the titles and abstracts of all articles that were found according to initial criteria. Subsequently, full-text articles of selected studies after the first selection were evaluated according to the final criteria, resulting in 17 original papers. The authors made all efforts to present the results of innovative and potentially valuable membrane modifications while considering clinical usefulness. The qualified papers included both in vitro and in vivo studies; clinical studies that were conducted on animal and human tissues were also selected. Over the selected 20-year period, an increase in the number of clinical trials and a tendency to conduct research on human tissues and the use of membrane modifications in vivo were noted.

## 4. Discussion

Studies that were included in this review were divided into three groups based on the type of membrane modification: antibiotic coating, ion-use modifications, and others. Table 1 presents the summary of obtained results, including information about membrane type, membrane material, type of modification, and additional properties.

### 4.1. Antibiotic Coating

Membranes that were coated with antimicrobial agents are a recently developed approach. The idea of using antibiotics on membranes for GTR/GBR has been based on observations of a detrimental effect of pathogens on periodontal healing in barrier membrane-assisted periodontal therapy, especially in the early stage of healing [37,38]. Currently, systemic antibiotics are commonly used for controlling pathogen growth after GBR. The use of local antibiotics could serve as an effective means for preventing harmful effects of periodontal pathogens, thereby limiting the systemic adverse reactions that are associated with such therapy. The addition of antibiotics to the membrane surface was initially aimed at reducing the level of pathogens [23,24]. However, it has been proven that antibiotics can provide additional benefits, such as a delayed collagen degradation, which helps in cell exclusion and the repopulation of progenitor cells [25].

Several antibiotics have been examined to enhance membrane properties. The most common are tetracyclines, because they are a group of broad-spectrum antibiotic agents with well-known efficacy and safety profile in the treatment of periodontal diseases [20]. Apart from antibacterial activity, tetracyclines exert anti-inflammatory, anti-collagenase, and wound-healing properties. They decrease bone resorption and inhibit osteoclast differentiation along with an enhancement of their apoptosis [25]. They inhibit matrix metalloproteinases, including collagenases, which play a role in damaging both connective tissue and bioabsorbable collagen membranes [25].

Most of the studies on antibiotic coatings are still in the experimental phase and they report the results of in vitro experiments or animal models. Lian et al. [22] described the use of bi-layered poly(lactic-co-glycolic) acid (PLGA) membrane with nanofibres that were loaded with doxycycline and dexamethasone. They observed an increased potential in osteogenic function in bone marrow stem cells of a rat, with an increase in alkaline phosphatase activity, calcium deposition, and up-regulation of osteocalcin. Kütan et al. reported better bone formation following the GBR procedure for collagen membranes that were treated with doxycycline than for those without any antibiotic treatment in another animal model that was infected with *Porphyromonas gingivalis* that could be present during GBR [21]. Zarkesh et al. conducted a clinical trial on humans using PTFE barrier membranes for treating intraosseous periodontal lesions. They found that tetracycline-coated PTFE barrier membranes improved periodontal attachment, which might be due to a significant decrease in periodontal pathogens on the implanted membranes during the early stage of healing [24]. In the literature, there are many other similar studies but without randomisation being conducted on small populations (therefore, they were excluded from this review and are not described).

The antibiotic coating seems to be the most desirable and most widely described GBR membrane modification. It is probably used because of a high risk of bacterial infection during this type of procedure. Modifications of barrier membranes with antibiotics have been successfully clinically achieved, but, due to the increasing clinical requirements regarding antibacterial properties, newer substances are being tested. To date, tetracycline seems to be the most promising antibiotic for this purpose.

### 4.2. Ion Modification

Enriching coating membranes with ions can significantly improve their biocompatibility and antibacterial properties. This could also prevent epithelial cell migration and enhance bone regeneration [39,40]. Metal and half-metal particles are the most commonly used ions in ion-membrane modification. These particles include silicon, titanium, and silver. Titanium and silver particles are used to mainly incorporate PLGA membranes. Silver particles are used to mainly incorporate chitosan membranes. Nanocomposite membranes can be incorporated with both silver and titanium.

The most promising technique of ion-membrane modification includes incorporation of SiO_2_ nanoparticles onto PO_2_-treated PLGA membrane. In an in vitro study, Castillo-Dali et al. investigated the properties of PLGA membranes after treatment with oxygen plasma and/or functionalising with nanometric layers of silicon (SiO_2_) or titanium (TiO_2_) dioxide particles. The research was conducted while using rabbit’s skulls. The authors reported that the oxygen plasma conditioning of membrane increased its roughness and promoted cell adhesion. It also improved the biodegradation ability of the barriers, which could be due to the effect of the combination of bone neoformation, mineralisation, resorption, and presence of osteoclasts and osteosynthetic activity. On the one hand, incorporated SiO_2_ stimulated osteosynthetic activity, which helped in bone formation. On the other hand, incorporated TiO_2_ induced a proper balance of the activity of osteoclasts and osteoblasts, which resulted in proper osteosynthesis. Thus, it can be concluded that PO_2_ conditioning helps in cell adhesion and SiO_2_ and TiO_2_ incorporation enhances osteosynthetic activity. A previous study suggested that the obtained results needed more accurate clinical studies that involve humans [28]. Castillo-Dali et al. (2017) again investigated the influence of cold plasma on PLGA membrane surface roughness and the incorporation of metallic-oxide particles on the osteoinductive capacity. They now considered larger bone defects than the previous one. Membranes that were conditioned with PO_2_ and then incorporated with SiO_2_ provided significantly higher bone regeneration and mineralised bone length. This kind of membrane also showed a better influence on osteoclast concentration and improved osteosynthetic activity [26]. It can be concluded that both SiO_2_ and TiO_2_ incorporation significantly influenced bone regeneration, and this can be a valuable direction of future clinical research on GBR membrane modification.

In addition, silver ions can be useful in GBR membrane modification. Silver nanoparticles have well-known antimicrobial properties and they can be used in many medical fields, including dentistry [16]. Jin et al. studied a novel type of biomimetic and bioactive silver ion-loaded calcium phosphate chitosan membrane. In this in vitro study, the authors used the electrospinning method to incorporate silver ion-loaded calcium phosphate into chitosan membrane, and then cross-linked it with vanillin. The membranes could release them in a continuous manner, providing better antibacterial properties due to silver ion incorporation. Silver ions are also released in a controlled manner, which at the same time ensure adequate antibacterial properties and a low level of cytotoxicity and, additionally, proper cytocompatibility. In authors’ viewpoint, this method could be successfully used in membrane modification; however, it requires additional clinical trials [27].

Some of the analysed studies investigated the properties of membranes that are incorporated with both silver ions and metallic-oxide particles [27,30]. Ye et al. conducted an in vitro study that investigates nanocomposite membranes incorporated with silver ions and TiO_2_. Incorporating silver ions improved antibacterial properties. The incorporated membranes showed good effects on the proliferation of osteoblast-like cells. Furthermore, this kind of membrane had sufficient biocompatibility and structure, which helped cell adhesion [27]. Zhang et al. conducted an in vivo study on rats and investigated the differences between expanded PTFE membrane and polyamide nanocomposite membrane that were modified with silver ions and TiO_2_. They showed that incorporated membranes demonstrated improved biocompatibility and similar osteogenic abilities. They also suggested that this kind of membrane could be a good direction for further development of GBR antibacterial membranes [29].

Some types of barrier membranes allow for modification with several types of ions at the same time. This results in the simultaneously improvement of several required parameters and a better therapeutic effect. The proper technique of ion incorporation also allows the control and prolonging of their release, which ensures the prolongation of the antibacterial properties of the membrane, for example, silver ions. These factors may indicate the superiority of ion incorporation over other modifications.

### 4.3. Other Modifications

Other modifications are related to membrane coating with alginate [31], hyaluronic acid [32], polyvinyl alcohol [34] and crystalline polypropylene (PP) [36], and subjecting the membranes to physical modifications, such as electron beam irradiation [33] and electrospinning [35].

#### 4.3.1. Alginate Coating

Alginate is a hydrophilic, biocompatible, anionic natural polymer, similar to extracellular matrices of tissues of living organisms, which allows for broad application in tissue regeneration [41,42,43,44,45] In the in vitro study that was conducted by Chen et al., chitosan membrane was coated with alginate-(1-4)-linked β-d-mannuronic acid (M units) and α-l-guluronic acid monomers. The membrane was prepared in a two-chamber-modified dialysis apparatus under constant stirring. A 0.05 wt % alginate was used in this procedure. An additional crosslinking agent was not needed, because of the electrostatic reaction between chitosan (cationic) and alginate (anionic). Electron spectroscopy verified the incorporation of alginate particles. Scanning electron microscopic observation revealed an increase in the roughness of the membrane surface in comparison to that of unmodified chitosan membrane. Further analysis revealed a decrease in water contact angle on the alginate-coated side from 88.4° to 34.2°, an increase in membrane water content to 65%, an increase in tensile resistance and stiffness, and a decrease in 3T3 fibroblast cell adherence to the alginate-coated side in comparison to that of the chitosan membrane side. In addition, in vitro degradation profile was examined. The membrane was immersed in phosphate-buffered saline at 37 °C with pH 7.4. After a 30-day trial, the membrane degenerated to 75% of its initial weight. 50% higher resistance to fibroblast cell adherence was achieved by modifying the membrane with alginate, thus providing more time to osteoblast cells to occupy the defected area [31].

#### 4.3.2. Hyaluronic Acid Coating

Hyaluronic acid (HA) is a polyanionic natural glycosaminoglycan. Due to its antibacterial properties and features, such as the promotion of cell migration and proliferation, it is used in various branches of medicine [46,47,48,49,50]. In the study that was conducted by Silva et al., HA use in GTR was assessed. The experiment was performed on rats. Two types of collagen membranes (porcine and bovine) were used. The membranes were soaked in 1% HA gel for 30 min before use. Features, such as inflammation response, foreign body reaction, signs of vascularisation, and hard and soft tissue regeneration, were investigated. There were no statistically significant differences in any of those aspects between the HA group and control group, thus showing that HA does not influence GTR [32].

#### 4.3.3. Polyvinyl Alcohol Coating

The most serious defects of chitosan membranes are considerable stiffness and hydrophobicity. Chitosan modification that was performed by Zhuang et al. using poly(vinyl alcohol) (PVA) was designed to overcome the above-mentioned disadvantages. The method that they developed consisted of synthesising chitosan with PVA polymer. PVA has high hydrophilic properties and excellent mechanical properties; it is also non-toxic and water soluble, and therefore it was chosen as the covering material. In addition, PVA-modified chitosan was obtained in different ratios of Chitosan/PVA, varying from 1:3 to 3:1.

Chitosan/PVA. Fourier-Transform Infrared Spectroscopy (FTIR), X-Ray Diffraction (XRD), and Differential Scanning Calorimetry (DSC) examined the biological, chemical, and mechanical properties of membranes in a laboratory. The Chitosan/PVA membrane was prepared by stirring a mixture containing 6% chitosan and 2% acetic acid water solution with PVA water solution I at different ratios. The solution was then heated up to 80 °C under constant mixing for 1 h. To ensure the removal of air bubbles, the solution was then left for 4 h. Afterwards, the mixture was poured in glass forms and then dried for 10 h under 50 °C. The glass plates with membranes were soaked with 5% NaOH to neutralise membrane pH, following which they were cleaned with distilled water and dried. FTIR spectra performed on Chitosan membranes and Chitosan/PVA membranes revealed compatibility between the Chitosan molecules and PVA. Chitosan and PVA had strong interactions with each other, creating hydrogen bonds between them. DSC thermograms showed that the PVA was able to increase the crystallinity of Chitosan/PVA membranes. As all of the membranes are used wet in practice GTR, tensile tests were carried out on wet condition to measure tensile strength. Furthermore, the addition of PVA to the Chitosan membrane can significantly modify its elasticity and wettability. The cytotoxicity tests indicated that Chitosan/PVA membranes have good biocompatibility. Adding PVA into Chitosan membranes modified its flexibility and wettability. Human osteosarcoma cells (MG63) that were used in a previous study were able to attach and spread on the membrane. The results of this study indicate the possibility to use this modification in future GTR technology [34].

#### 4.3.4. Crystalline Polypropylene Coating

Franco et al. have demonstrated a different approach to the subject of improving the characteristics of membranes. They assumed that increasing the hydrophobicity of barrier membranes would have a positive effect on the separation during the healing process in the GTR method. To achieve this, they used a porous PP membrane that was chemically treated to deposit a thin superhydrophobic coating [36]. It is well known that hydrophobicity results in smooth morphology of the surface. For smooth surfaces, such as Teflon, the maximal contact angle with water is 120°. Therefore, another method to improve the contact angle can be increasing porosity, such as in superhydrophobic substances. Erbil et al. reported a 160° contact angle to surface by increasing the porosity. The superhydrophobic coating was formed using PP and a selection of solvents and temperature to control surface roughness. A gel-like porous coating was obtained, which improved the contact angle of the surface. A possible conclusion of this study is that there is a correlation between porosity and the increasing contact angle. The higher the porosity, the more hydrophilic the surface will be [51]. Franco et al. used a similar concept, in which a chemically treated PP (CTPP) membrane was coated with methyl ethyl ketone and cyclohexanone. A thin layer of the hot solution of methyl ethyl ketone and cyclohexanone was deposited onto PP filter discs while using a spin coater programmed to rotate. The coated membranes were then immediately placed inside a vacuum oven to control PP crystallisation. They compared the coated membranes with widely available PTFE and PP membranes. Goniometer was used to measure the contact angle of the membrane with distilled water. Static, advancing, and receding contact angles were also identified. Scanning electron microscope (SEM) images were obtained to confirm these results. Furthermore, the experimental results showed that the untreated PP surface had a lower roughness than CTPP surface. Chemical treatment increased the hydrophobicity of the membrane. It also led to an increase in contact angle of new PP membrane when compared with PTFE membranes by 30° and by 42° when compared with the untreated PP membranes. An important fact of this study is the lack of biological and cytotoxic tests. Increased membrane thickness and the reduction of surface porosity are the possible side effects of treatment, which could be a huge disadvantage in some cases. Furthermore, membrane surface treatment could be a cheaper alternative to fluorinated membrane materials [36].

#### 4.3.5. Electron Beam Irradiation

Electron beam irradiation is a method in which linearly accelerated electrons interact with a surface, thus causing its damage [52,53]. The study conducted by Bilgi et al. reported the use of bacterial cellulose non-resorbable membrane irradiated at 100 or 300 kGy in GBR. Bacterial cellulose is a natural polymer that is produced by *Gluconacetobacter xylinus*. The authors showed that irradiation significantly reduces membrane mechanical properties, such as tensile strength and flexibility, and it enhances the adhesion, proliferation, and viability of fibroblast cells. In addition, in vitro degradation test showed an acceleration of membrane degradation in comparison to the non-irradiated material. The results of histological analysis showed that irradiated bacterial cellulose membrane achieved larger new bone gain than the non-irradiated membrane and collagen membrane. Higher bone growth was recorded while using 100 kGy than using 300 kGy irradiated membrane. In conclusion, optimal dose electron beam irradiation treatment resulted in the optimal rate of membrane biodegradation [33].

#### 4.3.6. Electrospinning

Electrospinning is another method of GBR membrane modification. It is a simple and versatile technique that is capable of making continuous fibres from a wide range of natural or synthetic polymers, such as chitosan [54]. This technique also allows for fabricating scaffolds that can mimic extracellular matrix [35,54]. Therefore, electrospinning can also be used for creating GBR membranes. Qasim et al. used the electrospinning technique to modify the orientation of chitosan fibres. They obtained highly aligned and randomly oriented fibre configuration and performed a series of laboratory tests. Cell cultures were developed for analysing the viability of osteoblastic cells on different fibre orientations. They used human osteosarcoma cells (MG63) and human embryonic stem cell-derived mesenchymal progenitor cells (hES-MPs). Cell viability significantly increased for both types of cells over time for each type of fibre orientation, but calcium deposition was only higher for random fibre membranes. A statistically significant difference occurred between days 14 and 28. The results demonstrated that randomly oriented fibres promote osteoblastic cell proliferation and that aligned fibres promote ligament growth [35]. Strength tests were carried out on dry and wet membranes. Dry-aligned fibres showed a significantly higher modulus (E) than dry random fibres. The electrospun fibres were the strongest when compared with other commercially available resorbable GTR membranes [55]. Dry-aligned fibres exhibited a tensile strength of 28.76 MPa, which was higher than that of collagen GTR membranes [55,56]. In conclusion, both orientation types of chitosan fibre membranes can be used in GTR and they have the ability to mimic local tissue areas.

The most frequently modified membranes among the included studies were the chitosan ones. One of the most serious defects of unmodified chitosan membranes is low roughness. In accordance with the studies presented in this review, alginate coating can be a promising technique of increasing chitosan membrane roughness. Stiffness and hydrophobicity are other serious disadvantages of this kind of membranes. These can be successfully overcome by applying PVA coating that increases their flexibility and wettability. The antimicrobial capacity of chitosan membranes cannot be so easily changed. Although HA coating is promising, it does not significantly affect the antibacterial capacity of the chitosan membranes. Another modifiable aspect of chitosan membranes is the cross-linking of fibres. Electrospinning technique seems to be the most promising technique to modify the orientation of chitosan fibres. In this case, electrospinning is mainly aimed to strengthen the fibre structure and make it mimic natural human tissues.

## 5. Conclusions

The results of the included studies on the modified barrier membranes seem to be promising in terms of both safety and benefits for patients. Modifications result in a large spectrum of effects. Coating with antibiotics lowers the probability of bacterial infection and promotes cell growth. Enriching membranes with ions significantly improves their biocompatibility and antibacterial properties; it can also prevent epithelial cell migration and enhance bone regeneration. Alginate coating increases the wettability and mechanical properties of membranes. Coating with PVA improves the mechanical properties. Coating with crystalline PP enhances the separation between soft and bone tissue. Irradiation accelerates biodegradation and decreases mechanical properties. Electrospinning increases the proliferation of osteoblastic cells and enhances tissue regeneration.

Further clinical studies are needed on a large group of patients to clearly confirm the effects observed in animal and in vitro studies to determine the most effective dose of antibiotic agents, irradiation, and ions, the most efficient antibiotics for specific membrane materials, and the most suitable way of coating to achieve the best membrane properties before they can be introduced to routine clinical practice.

## Figures and Tables

**Table 1:** Summary of included studies.

Author, year [ref.]	Type of Membrane	Morphology of Membrane	Membrane Material	Modification	Additional Properties
Jin, et al., 2014 [20]	Resorbable membrane	Compact	Silk fibroin solution casted on a polystyrene dish	Impregnation with tetracycline	Increase in proliferation, osteogenic potential of gingiva-derived mesenchymal stem cells
Kütan, et al., 2016 [21]	Resorbable membrane	Not reported	Collagen	Impregnation with doxycycline	Inhibition of bacterial growth
Lian, et al., 2019 [22]	Resorbable bi-layered composite membrane	Porous	Poly(lactic-co-glycolic) acid	Poly(lactic-co-glycolic) acid nanofibres loaded with doxycycline and dexamethasone	Inhibition of bacterial growth
Ma, et al., 2016 [23]	Resorbable asymmetric membrane	Porous	Collagen, chitosan	Minocycline-loaded chitosan nanoparticles	Inhibition of bacterial growth, promotion of osteoblast and fibroblast growth, promotion of angiogenesis
Zarkesh, et al., 1999 [24]	Non-resorbable membrane	Porous	Polytetrafluoroethylene	Impregnation with tetracycline	Reduced colonisation of membranes with periodontal pathogens
Zohar, et al., 2004 [25]	Resorbable membrane	Not reported	Collagen	Impregnation with tetracycline	Slowing membrane degradation
Castillo-Dali, et al., 2017 [26]	Resorbable membrane	Not reported	Poly(lactic-co-glycolic) acid	Incorporation of bioactive layers of SiO_2_ onto poly(lactic-co-glycolic) acid membranes modified with PO_2_	Enhance bone regeneration, stimulation of adhesion of osteogenic mediators and cells, stimulation of new bone formation, mineralisation enhancement of osteosynthetic activity
Jin, et al., 2018 [27]	Resorbable membrane	Porous	Chitosan	Electrospun silver ion-loaded calcium phosphate subsequently crosslinked with vanillin	Inhibition of bacterial growth, increased biocompatibility
Castillo-Dali, et al., 2014 [28]	Bilayer resorbable membrane	Not reported	Poly(lactic-co-glycolic) acid	Poly(lactic-co-glycolic) acid being treated with oxygen plasma (PO_2_) and/or being functionalised with silicon dioxide (SiO_2_) or titanium dioxide (TiO_2_) nanoparticles	Enhanced osteosynthetic activity, enhanced bone regeneration
Zhang, et al., 2010 [29]	Resorbable composite membrane	Porous	Polyamide nanocomposite membrane	Silver-hydroxyapatite/titania	Increased biocompatibility, increased antibacterial properties, induced inflammatory response, enhanced bone regeneration
Ye, et al., 2011 [30]	Resorbable composite membrane	Porous	Polyamide nanocomposite membrane	Silver–hydroxyapatite/titania	Increased adhesion, increased proliferation of osteoblast-like -cells
Chen, et al., 2006 [31]	Resorbable membrane	Not reported	Chitosan	Alginate coating	Increased wettability, increased stiffness, increased tear strength, increased resistance to fibroblast cell adhering
Silva, et al., 2017 [32]	Biodegradable bovine and porcine membrane	Porous	Collagen	Impregnation with hyaluronic acid	No modifying effect on guided bone regeneration
Bilgi, et al., 2016 [33]	Non- resorbable membrane	Not reported	Bacterial cellulose	Electron beam irradiation	Acceleration of degradation, reduction of mechanical properties
Zhuang, et al., 2012 [34]	Resorbable membrane	Not reported	Chitosan	Synthesise with poly(vinyl alcohol)	Increased tensile strength in wet conditions
Qasim, et al., 2017 [35]	Resorbable membrane	Not reported	Chitosan	Electrospinning	Increased proliferation of osteoblastic cells, enhanced tissue regeneration for all fibre types, randomly oriented fibre promote osteoblastic cell proliferation, aligned fibres promote ligament growth
Franco, et al., 2008 [36]	Non-resorbable, synthetic polymer	Porous	Polypropylene, polytetrafluoroethylene	Porous crystalline polypropylene coating	Enhanced separation of repaired bone and soft tissue
